# Internet and Computer-Based Cognitive Behavioral Therapy for Anxiety and Depression in Youth: A Meta-Analysis of Randomized Controlled Outcome Trials

**DOI:** 10.1371/journal.pone.0119895

**Published:** 2015-03-18

**Authors:** David Daniel Ebert, Anna-Carlotta Zarski, Helen Christensen, Yvonne Stikkelbroek, Pim Cuijpers, Matthias Berking, Heleen Riper

**Affiliations:** 1 Leuphana University, Innovation Incubator, Division Health Trainings Online, Lueneburg, Germany; 2 Friedrich-Alexander University Nuremberg-Erlangen, Department of Psychology, Clinical Psychology and Psychotherapy, Erlangen, Germany; 3 Black Dog Institute, Sydney, Australia; 4 Center for Child and Adolescent Studies, University of Utrecht, the Netherlands; 5 GGZ inGeest, Regional Mental Health Service Centre, VU University Medical Centre, Amsterdam, the Netherlands; 6 Department of Clinical Psychology and EMGO Institute for Health and Care Research, VU University, Amsterdam, the Netherlands; Merced, UNITED STATES

## Abstract

**Background:**

Anxiety and depression in children and adolescents are undertreated. Computer- and Internet-based cognitive behavioral treatments (cCBT) may be an attractive treatment alternative to regular face-to-face treatment.This meta-analysis aims to evaluate whether cCBT is effective for treating symptoms of anxiety and depression in youth.

**Methods and Findings:**

We conducted systematic searches in bibliographical databases (Pubmed, Cochrane controlled trial register, PsychInfo) up to December 4, 2013. Only randomized controlled trials in which a computer-, Internet- or mobile-based cognitive behavioral intervention targeting either depression, anxiety or both in children or adolescents up to the age of 25 were compared to a control condition were selected. We employed a random-effects pooling model in overall effect analyses and a mixed effect model for sub-group analyses. Searches resulted in identifying 13 randomized trials, including 796 children and adolescents that met inclusion criteria. Seven studies were directed at treating anxiety, four studies at depression, and two were of a transdiagnostic nature, targeting both anxiety and depression. The overall mean effect size (Hedges’ g) of cCBT on symptoms of anxiety or depression at post-test was g=0.72 (95% CI:0.55-0.90, numbers needed to be treated (NNT)=2.56). Heterogeneity was low (I²=20.14%, 95% CI: 0-58%). The superiority of cCBT over controls was evident for interventions targeting anxiety (g=0.68; 95% CI: 0.45-0.92; p < .001; NNT=2.70) and for interventions targeting depression (g=0.76; 95% CI: 0.41-0.12; p < .001; NNT=2.44) as well as for transdiagnostic interventions (g=0.94; 95% CI: 0.23-2.66; p < .001; NNT=2.60).

**Conclusions:**

Results provide evidence for the efficacy of cCBT in the treatment of anxiety and depressive symptoms in youth. Hence, such interventions may be a promising treatment alternative when evidence based face-to-face treatment is not feasible. Future studies should examine long-term effects of treatments and should focus on obtaining patient-level data from existing studies, to perform an individual patient data meta-analysis.

## Introduction

Depression and anxiety are common in children and adolescents (in the following referred to collectively as *youth*) [1–3] and often co-occur [4, 5]. Both disorders are associated with substantial burdens [6, 7] an increased risk for other mental disorders [8–10] and they often tend to persist in adulthood [11–13].

In the past decades, a number of psychological treatments for anxiety and depression in youth have been developed with demonstrated efficacy in a substantial number of clinical trials [14–18]. Cognitive behavioral therapy (CBT) is generally regarded as the treatment of choice for depression and anxiety in youth [19].

However, up to 80% of children and adolescents with mental health needs receive no treatment [20–24]. The reasons include not only a lack of treatment availability, but also reticence to seek help because of perceived stigma associated with mental illness, discomfort discussing mental health problems, and / or a preference for self-help [25].

Using the computer or the Internet to provide CBT may overcome some of the limitations of traditional treatment services. Advantages of computer and Internet-based CBT (cCBT) include (1) availability (2) anonymity, (3) accessibility at any time and place, (4) flexibility in self-direction and self-pacing, (5) reduced travel time and costs for both participants and clinicians, and (6) the appeal of interactivity and visual attractiveness of Internet-based programs [26, 27, 28]. Given the Internet savviness of the younger generations, these advantages might be even more relevant in youths than in adults.

cCBT is effective in the treatment of depressive [29] and anxiety disorders [30] in adults. For example, a systematic review of 19 randomized controlled trials evaluating Internet-based and other computerized interventions for adult depression symptoms in N = 2996 individuals found a mean effect size of d = 0.56. In another meta-analyses on self-help interventions for anxiety disorders, a pooled mean effect size of d = .90 was found across 27 randomized controlled trials evaluating Internet-based treatments for anxiety [31].

Less is known, however, about the effectiveness of cCBT for anxiety and depression in youth. In a recent systematic review [28], only three randomized controlled trials were included. Hence, the authors could not calculate pooled effect sizes using meta-analytic techniques. With the recent addition of several new published trials, we performed a meta-analysis to evaluate the efficacy of cCBT for anxiety and depression in youth (up to the age of 25) in comparison to a non-active control condition within randomized controlled trials.

## Methods

### Study selection

**Fig 1:**
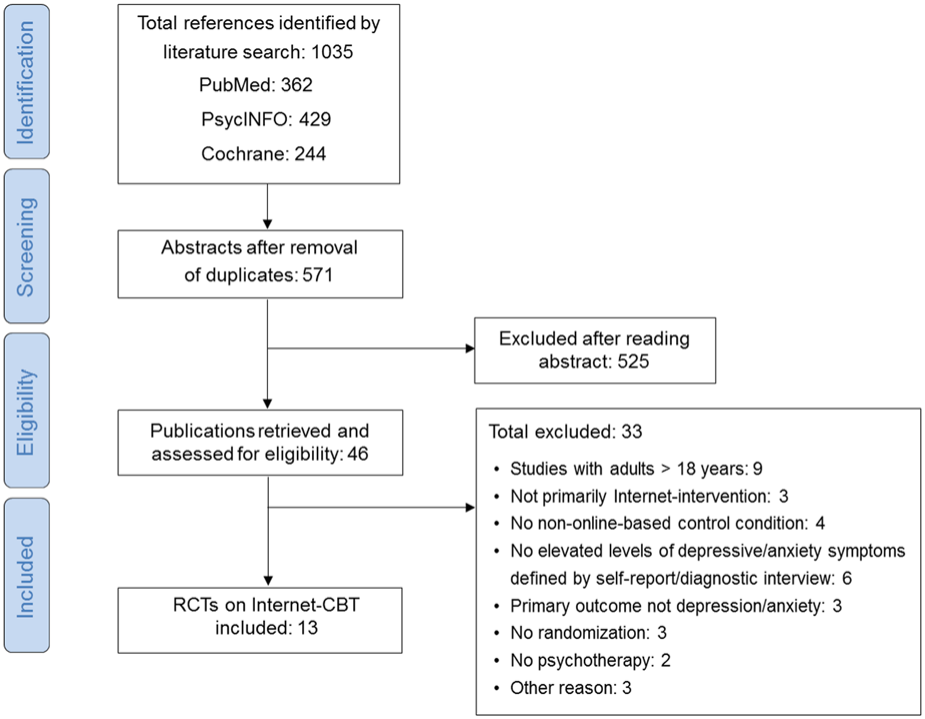
Flow Chart.

Several strategies were used to identify relevant studies. Two independent assessors (Ebert & Zarski) searched three major bibliographical databases (PubMed, PsycInfo, Cochrane) by combining terms indicative of each of the disorders with terms indicative of psychological treatment and randomized controlled trials. The searches were performed December 04, 2013. We also checked the references of the identified studies for earlier publications. Details of the searches are provided in Fig. 1.

We included (a) randomized controlled trials in which (b) a computer or Internet- or mobile-based (c) cognitive behavioural intervention (d) targeting either depression, anxiety or both (e) in children or adolescents up to the age of 25 (f) were compared to a non-active control condition (wait-list; placebo). We only included studies (g) that used an anxiety or depression measure as the primary outcome measure and (h) in which participants were selected because of elevated levels of depressive/anxiety symptoms based on either a standardized self-report measure or a formal diagnosis of major depressive or any anxiety disorder (including OCD), and (i) effect sizes could be calculated from the information provided in the paper. If effect sizes could not be calculated, authors were contacted to retrieve the necessary information. Studies were not included when they had only an active treatment comparison (e.g., face-to-face CBT) or only included participants older than 18 years. Comorbid general medical and psychiatric or substance/alcohol use disorders were not used as an exclusion criterion. No language restrictions were applied. Studies that evaluated interventions in which the Internet/computer was not the primary treatment modality (e.g., blended treatments with face-to-face psychotherapy and some online elements) were excluded.

### Risk of bias assessment and data extraction

Risk of bias assessment and data extraction was conducted independently by two assessors (Ebert & Zarski). We assessed the validity of included studies using four criteria of the *Risk of Bias* assessment tool, developed by the Cochrane Collaboration [32] (adequate generation of allocation sequence, the concealment of allocation to conditions, masking of assessors, and the missing data handling (the assessment was positive when intention-to-treat analyses were conducted)). We examined the relationship between risk of bias and the effect size by performing meta-regression techniques. In these analyses, the total bias score was entered as the dependent variable. We compared effect sizes of studies rated as low-risk of bias (fulfilling all four assessed criteria) compared to studies with some risk of bias (fulfilling less than four assessed criteria). We also identified and coded additional aspects of the included studies, relating to the study design, participants and interventions.

### Study design

Variables related to the study design included the target condition (anxiety vs. depression vs. transdiagnostic) and the comparison condition (no treatment vs. placebo). The comparison group was coded as placebo control group if cCBT was compared to an active condition with no intended therapeutic properties that would be expected to produce change in the target diagnosis [33].

We also coded the outcome informant (youth, parent, teacher), that is, whether the information about symptoms were derived from the youth report, parent report, or teacher report. We further coded the type of outcome assessment (self-report vs. observer based) and whether the studies included a follow-up (yes/no; duration).

### Participant characteristics

In line with previous meta-analyses on psychotherapy in children and adolescents [16, 34, 35], we classified studies as *child*, *adolescents* or *mixed* studies. Studies in which all participants were 13 years-old or younger were classified as *child*, studies in which all participants were over 13 years-old were classified as *adolescent*, studies that included both children and adolescents were classified as *mixed*. The mean age was also coded, and the percentage of boys was included. We further coded whether studies included only participants with a confirmed diagnosis of depression or an anxiety disorder using an established diagnostic interview (yes/no).

### Intervention characteristics

Interventions were classified as with or without parental involvement (yes/no) and with or without professional guidance (therapeutic or simply administrative) (yes/no). We also coded the technology on which the intervention was delivered (computer: cCBT; Internet: iCBT; mobile: mCBT), the setting in which the intervention was conducted (at home/not at home, e.g., clinic, school, university), as well as the number of treatment sessions.

### Meta-analysis

For each comparison between cCBT for anxiety or depression or both and a control group, we calculated the effect size indicating the difference between the two groups in change from baseline to post-test (Hedges’ g). To calculate pooled mean effect sizes, we used the Comprehensive Meta-Analysis (CMA) software (Biostat, Inc.). In the main outcome analysis, we examined the overall mean effects of cCBT on symptoms of depression or anxiety. To generate a mean effect size, we only used instruments that related to the principal measure of the disorder. If interventions were transdiagnostic, targeting both anxiety and depression, we used the pooled effect size of these outcomes. If more than one measure per outcome was used, the mean of the effect sizes was calculated, so that each study provided only one effect size. Given that youth depression and anxiety symptoms highly correlate [4, 5] and in light of the current debate over whether depression and anxiety require separate treatments or can be treated by combined interventions [16, 36], we also examined the overall effects of all interventions on symptoms of anxiety and depression in a separate analysis.

We expected considerable heterogeneity among the studies, hence we employed a random-effects pooling model (DerSimonian-Laird) in all analyses. Given the difficulty of interpreting the standardized mean difference (Hedges’ g) from a clinical perspective, we transformed these values into the number-needed-to-treated (NNT) using the formula provided by Kraemer and Kupfer [37]. The NNT indicates the number of patients that must be treated to generate one additional positive outcome [38].

As a test of homogeneity of effect sizes, we calculated the *I* ^*2*^ statistic as an indicator of heterogeneity in percentages [39, 40]. We calculated 95% confidence intervals around *I²* [39] using the non-central chi-squared-based approach within the heterogi module for Stata [41]. We also calculated the Q statistic but only reported whether the result was significant.

Publication bias was tested by visually inspecting the funnel plot on primary outcome measures. We also conducted Egger’s test to quantify the bias captured by the funnel plot and to test whether it was significant [42]. The Duval and Tweedie trim-and-fill analysis [43] was performed to further verify an unbiased estimate of the pooled effect size. This method calculates an estimation of the number of missing studies and the effect that these studies might have had on its outcome.

We further conducted a series of subgroup-analyses, according to the mixed-effect model [44]. In this model, studies within subgroups are pooled with the random-effects model, while tests for significant differences between subgroups are conducted with the fixed-effects model.

## Results

### Selection and inclusion of studies

Thirteen studies met inclusion criteria for the meta-analysis of cCBT for depression and anxiety in youth. Fig. 1 presents a PRISMA flowchart describing the inclusion process. Two reviewers (Ebert & Zarski) independently reviewed studies for inclusion or exclusion. Agreement between raters on inclusion was 100%.

### Characteristics of included studies

The 13 studies on cCBT for symptoms of anxiety and depression in youth included 796 participants, 420 in the treatment groups and 376 in the control groups. Selected characteristics of the included studies are displayed in Table 1. Seven studies were directed at treating anxiety, four at depression, two were transdiagnostic and targeted both depression and anxiety. Two studies were aimed at children (< 13 years), six studies at adolescents (≥ 13 years), five studies had a mixed sample. About half of the studies (n = 6) included only participants with a diagnosis, confirmed by an established diagnostic interview. The intervention in the majority of studies could be completed in the home of participants (n = 9).

**Table 1:** Selected characteristics of randomised controlled studies examining the effects of cCBT and iCBT for anxiety and depression in children and adolescents.

Study	Cond	Age Range	Inclusion	Diagn	Recr	Setting	Mean Age (SD), % Boys	Conditions	N	N_mod_	Par Inv	Guid-ance	Outcomes and Method	FU	Bias^a^	Country
Fleming,	Dep	13–16	CDRS-R >29, excl: severe depr	N	School	School	14.9 (0.79) 56	cCBT (SPARX)	20	7	N	Y	D: CDRS-R (Y, O), RADS-2 (Y, SR); A: SCAS-C (Y, SR)	N	++++	NZ
2012 [47]								WL	12							
Keller,	Anx	6–12	GAD > 8, SepA > 4 / SP > 7 on SCARED-P, excl:depr T-score > 65 on CDI-S	N	Com	Home	8.41 (1.59), 51.35	iCBT	22	12	Y	Y	A: SCARED-P (P, SR), DISC IV-P (P, O), RCMAS (Y, SR); D: CDI-S (Y, SR)	N	++++	USA
2009 [48]																
								WL	15							
Khanna, 2010 [49]	Anx	7–13	SepA, SoP, GAD, SP / PD on ADIS-P CSR > 3	Y	Com	Uni	10.1 (1.6), 67.35	cCBT (Camp Cope-A-Lot)	17	12, Par:2	Y	Y	A: ADIS-P CSR (P, O), MASC (Y, SR)	N	+?++	USA
								Placebo	16				D: CDI (Y, SR)			
Makarushka, 2012 [46]	Dep	11–15	CES-D > 15, excl: no MD, D / M on K-SADS-E	Y	Com	Home	12.7 (1.24) 44	iCBT (Blues Blaster)	76	6	N	N	D: CES-D (Y, SR)	6M	+?++	USA
								Placebo	85							
March,	Anx	7–12	Anx Dis (other than OCD, PD / PTSD) on ADIS-C/-P CSR > 3	Y	Com	Home	9.45 (1.37), 51.95	iCBT, (BRAVE for Children—ONLINE)	40	10 + 2 Boost Par:6	Y	Y	A: ADIS-C (Y, O), /-P CSR (P, O),SCAS-C (Y, SR), /-P (P, SR), D: CES-D (Y, SR)	N	++++	AU
2009 [50]																
								WL	33							
Sethi,	Trans	15–25	low/ moderate depr/anx on DASS-21, excl: severe Depr/Anx	N	School	Uni	19.47 (1.57) 34.21	iCBT	9	5	N	Y	D+A: DASS-21 (Y, SR), K, K10 (Y, SR)	N	??+?	AUS
2010 [51]																
								No treatment	10							
Spence,	Anx	12–18	GAD, SoP, SepA, SP on ADIS-C/-P CSR > 3, excl: prim diag of PD, OCD/ PTSD, moderate—severe mood disturbance on ADIS-C > 5	Y	Com	Home	13.98 (1.63), 40.87	iCBT, BRAVE for Children—ONLINE	44	10 + 2 Boost Par: 5 + 2 Boost	Y	Y	A: ADIS-C (Y, O) /-P CSR (P, O), SCAS-C (Y, SR), /-P (P, SR)	N	++++	AU
2011 [52]																
								WL	27							
Stallard,	Trans	11–16	GAD, SP, SoP / PD, excl: PTSD; mild/moderate depr, excl: severe depr	N	Clin	Home	Median 12, 66.7	cCBT (Think, Feel, Do)	10	6	N	Y	A: SCAS (Y, SR), D: AWS (Y, SR)	N	+++-	UK
2011 [53]																
								WL	10							
Stasiak,	Dep	13–18	CDRS-R > 29, RADS-2 > 75	N	School	School	15.2 (1.6) 59	cCBT (The Journey)	17	7	N	N	D: CDRS-R (Y, O), RADS-2 (Y, SR)	1M	++++	NZ
2012 [45]								Placebo	17							
Storch, 2011 [54]	Anx	7–16	OCD on ADIS-IV-C/-P CSR > 3, CY-BOCS > 15	Y	Clin Com	Home	11.10 (2.59), 61	cCBT WL	16 15	14	Y	Y	A: ADIS-IV-C (Y, O) /-P CSR (P, O), CY-BOCS (Y, O), COIS-C (Y, SR) /-P (P, SR), MASC (Y, SR),	N	+?-+	USA
													D: CDI (Y, SR)			
Tillfors,	Anx	15–21	SoP on SPSQ-C, public speaking fears, Depr < 30 on MADRS-S	Y	Com	Home	16.5 (1.6), 10.53	iCBT	10	9	N	Y	A: LSAS-SR (Y, SR); SPS (Y, SR), SIAS (Y, SR), SPSQ (Y, SR), BAI (Y, SR); D: MADRS-S (Y, SR)	N	??+-	SWE
2011 [55]																
								WL	9							
Wuthrich, 2012 [56]	Anx	14–17	Any Anx Dis on ADIS-IV-C/-P CSR	Y	Com	Home	15.17 (1.11) 37.21	cCBT, Cool TeensWL	24 19	8	Y	Y	A: ADIS-C (Y, O) /-P CSR (P, O), SCAS-C (Y, O) /-P (P, O)	N	++++	AU
Van der Zanden,	Dep	16–25	CES-D >10 < 45	N	Com	Home	20.9 (2.2), 15.6	iCBT, Master your Mood	121	6	N	Y	D: CES-D (Y, SR), A: HADS-A (Y, SR)	N	++++	NL
2012 [57]								WL	123							

^a)^ Bias: Risk of Bias: In this column a positive or negative sign is given for four risk of bias criteria, respectively: allocation sequence; concealment of allocation to conditions; blinding of assessors; and intention-to-treat analyses. A? is given if there were not enough information to judge.

Abbreviations:

ADIS-C: Anxiety Disorders Interview Schedule for Children; ADIS-IV-C: for DSM-IV: Child Version; ADIS-IV-P: Parent Version; ADIS-P: Parent Version; Anx: Anxiety; Attention: Attention Control; AU: Australia; AWS: Adolescent Well Being Scale; BAI: Beck Anxiety Inventory; Boost: Booster Sessions; CBT: Cognitive Behaviour Therapy; cCBT: Computerized CBT; CDI-S: Children´s Depression Inventory, Short Form; CDI: Children's Depression Inventory; CDRS-R: Children’s Depression Rating Scale, Revised; CES-D: Center for Epidemiologic Studies Depression Scale; Clin: Clinical Sample; COIS-C: Child Obsessive Compulsive Impact Scale, Child Version; COIS-P: Parent Version; Com: Community Sample; Cond: Condition; CSR: Clinical Severity Rating; CY-BOCS: Children's Yale-Brown Obsessive-Compulsive Scale; D: Dysthymia; DASS-21: Depression Anxiety Stress Scale Dep: Depression; Diagn: Diagnostic Confirmation, Diagnostic Interview + all participant fulfil disorder (no subclinical); Dis: Disorders; DISC IV-P: Diagnostic Interview Schedule for Children; Excl: Excluded; FU: Follow-Up; GAD: Generalized Anxiety Disorder; Guidance: Therapeutic or Administrative Support by a Professional; HADS-A: Hospital Anxiety and Depression Scale (Anxiety subscale); iCBT: Internetbased CBT; K-SADS-E: Schedule for Affective Disorders and Schizophrenia for School-Age Children, Epidemiological Version; K10: Kessler Psychological Distress Scale; LSAS-SR: Liebowitz Social Anxiety Scale, Self-Report; M: Month: Trans: Transdiagnostic; M. Mania; MADRS-S: Montgomery-Åsberg Depression Rating Scale-Self-Rated; MASC: Multidimensional Anxiety Scale for Children; MD: Major Depression; N: NoN: Number of participants in each condition;NL: Netherlands;N_mod_: Number of modules in the intervention;NZ: New Zealand; O: Observerrating (Clinician) OCD = Obsessive Compulsive Disorder; P: Parent Report; Par Inv: Parental Involvement;Par: Parent Sessions; PD: Panic Disorder; Prim Diag: Primary Diagnosis; PTSD: Posttraumatic Stress Disorder; RADS-2: Reynolds ´Adolescent Depression Scale, 2^nd^ Edition; RCMAS: Revised Children ´s Manifest Anxiety Scale; Recr: Recruitment; SCARED-P: Screen for Child Anxiety Related Emotional Disorders, Parent Version; SCAS-C: Spence Children´s Anxiety Scale, Child Version; SCAS-P: Parent Version; SD: Standard Deviation, SepA: Separation Anxiety Disorder; SIAS: Social Interaction Anxiety Scale; SoP: Social Phobia; SP: Specific Phobia; SPS: Social Phobia Scale; SPSQ-C: Social Phobia Screening Questionnaire for Children; SR: Self Report; SWE: Sweden; UK: United Kingdom; Uni: University Lab; USA: United States of America; WL: Waiting List Control; Y: Yes; Y: Youth Report.

A non-treatment comparison was used in 10 studies, the other three studies applied a placebo control. The majority relied on a multi-method outcome assessment approach (i.e., observer-based outcome interview and self-report measure, n = 8). Only five studies used only self-report measures. Outcome informants were both youths and parents in seven studies and youth-only in six studies. Studies using youth-only as outcome informant were mainly directed at adolescents (n = 4), two studies on mixed-age range samples. Many studies included a follow-up assessment. However, only two studies assessed a follow-up in both the intervention and control group [44, 45]. Thus, we were not able to examine long-term effects of treatments.

In most studies, the intervention was delivered via the Internet (n = 8), followed by computer (n = 5). The number of treatment sessions ranged from 6 to 14. Six studies were classified as brief treatment (6–7 sessions) and seven as long treatment (≥ 8 sessions). Parents were involved in the intervention within six studies and the majority of interventions included guidance by a professional (n = 11). Four Studies were conducted in the USA and Australia, respectively, two studies were conducted in New Zealand, two studies in Sweden and one study was conducted in the UK and the Netherlands, respectively. Thus, all studies were conducted in high-income countries. Cohen's kappa for agreement between independent raters was very good (κ = .84).

### Risk of bias

Overall risk of bias was low. Eleven studies reported an adequate sequence generation. Twelve studies reported blinding of outcome assessors or used only self-report outcomes, whereas five did not report blinding. Only eight studies reported the information needed to clarify whether allocation to conditions was performed by an independent (third) party. Ten studies reported adequate handling of missing data, using intention-to-treat principles. Seven studies met all four quality criteria, four studies met three of four criteria, and two studies met only one criterion. Inter-rater reliability between independent raters on the risk of bias was very good (κ = 0. 94).

### Effects of cCBT on symptoms of anxiety/depression when compared to a control group

**Fig 2:**
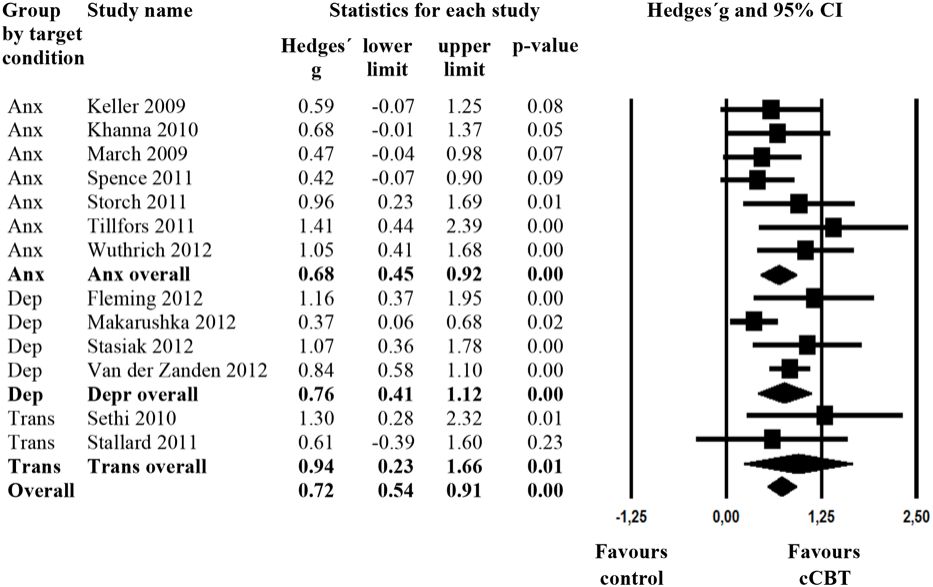
Forest Plot.

The overall mean effect size of cCBT on symptoms of anxiety or depression when compared to a control group at post-test was g = .72 (95% CI: 0.55 to 0.90; p <. 001; Table 2). This value corresponded to the number needed to be treated to achieve one additional positive outcome of 2.56. Heterogeneity was low (I² = 20.14%, 95% CI: 0% to 58%). Fig. 2 provides a forest plot of the effect sizes per study and the corresponding 95% CI.

**Table 2:** Effects of computer and internet-based CBT for anxiety and depression in youth when compared with control groups at post-test: Hedges’ g.

		N_co_	g	95% CI	Z	I^2^	P ^a^	NNT
Overall effect (primary outcome)		13	0.72	0.55~0.90	8.10***	20.14 (0~58)		2.56
Highest effect size removed		12	0.70	0.53~0.87	8.11***	15.11 (0~55)		2.63
Lowest effect size removed		12	0.80	0.64~0.96	9.65***	0 (0~58)		2.34
Only studies with all participants ≤18		10	0.61	0.43~0.80	6.52***	4.55 (0~64)		2.99
Overall on depressive symptoms		11	0.56	0.31~0.82	4.34***	53.88 * (9~77)		3.25
Overall on anxiety symptoms		10	0.65	0.40~0.90	5.13***	52.09 ^O^ (0~72)		2.82
Study characteristics								
Target condition	Anxiety	7	0.68	0.45~0.92	5.68***	0 (0~71)	.77	2.70
	Depression	4	0.76	0.41~1.12	4.21***	61.42^O^(0~87)		2.44
	Transdiagnostic	2	0.94	0.23~1.66	2.60***	0 ^c^		2.02
Age group	Children	3	0.56	0.21~0.91	3.13***	0 (0~90)	.007	3.25
	Adolescents	6	0.95	0.76~1.17	9.22***	0 (0~75)		2.01
	Mixed	4	0.46	0.22~0.70	3.75***	0 (0~85)		3.91
Confirmation of Disorder	Confirmed diagnosis	6	0.71	0.44~0.99	5.07***	13.67 (0~78)	.75	2.63
	Anxiety/depressive symptoms	7	0.74	0.49~1.00	5.70***	34.99 (0~73)		2.50
Risk of bias score	Low (4)	7	0.77	0.59~0.95	8.34***	0 (0~71)	.97	2.42
	Some risk (< 4)	6	0.72	0.37~1.07	4.06***	33.55 (0~73)		2.56
Intervention characteristics								
Parental Involvement	No	7	0.83	0.53~1.13	5.47***	45.40 ^O^ (0~77)	.33	2.26
	Yes	6	0.64	0.40~0.88	5.14***	0 (0~77)		2.86

Note: N _comp_, Number of comparison;

^a^ This p values indicate weather differences between subgroups are significant

^c^ calculation of 95% CI not possible because dfs are 1.

^O^: p <. 1;

* p <. 05;

*** p <. 001.

Excluding studies that also assessed participants above 18 years of age [51, 57, 58] resulted in an effect size of 0.61 (95% CI: 0.55 to 0.90). Excluding the study with the lowest effect size [46] further reduced heterogeneity (I² = 0, 95% CI: 0% to 58%), while excluding the study with the highest effect size [51] did not change heterogeneity substantially (I² = 15.11%, 95% CI: 0% to 55%).

Including also anxiety outcomes of interventions targeting depression, the effect size on anxiety was g = 0.65 (95% CI 0.40 to 0.90) with an NNT of 2.82. Including also depression outcomes of interventions targeting anxiety resulted in a pooled effect size of g = .56 (95% CI 0.31 to 0.82), NNT = 3.25.

### Subgroup and moderator analyses

We conducted a series of subgroup analyses (Table 2). Effects in subgroups were all in favor of cCBT and all significantly different from zero. Moderator analyses revealed that age group significantly moderated treatment outcome, with studies aimed at adolescent achieving better results (g = 0.95, 95% CI 0.76 to 1.17) compared to studies aimed at children (g = 0.51, 95% CI: 0.11 to 0.92) and mixed samples (g = 0.48, 95% CI 0.25 to 0.71). Given that some meta-analyses on treatments of anxiety disorders in youth [15] categorized children up to 13 years of age as children, we repeated the analysis classifiyng the study of Khanna [49] as directed at children. This result did not produce a different outcome pattern, with slighty higher effects for children (g = 0.56, 95% CI 0.21 to 0.91, NNT = 3.25) but still a significant difference in effectiveness between age groups (p = .007). We found that target condition, confirmation of disorder using a diagnostic interview, parental involvement, or risk of bias was not significantly associated with the size of the effect (Table 2).

### Publication bias

The inspection of the funnel plot and Egger’s test indicated some possible publication bias. However, after adjustment for missing studies using the Duval-Tweedie trim and fill procedure (four imputed studies) Hedge’s g for the main outcome analyses was 0.64 (95% CI 0.46 to 0.82), corresponding to an NNT of 2.86.

## Discussion

We found cCBT for youth was associated with significant and moderate to large effects on symptoms of anxiety and depression, with an NNT of 2.56. Overall, risk of bias was low and the seven studies that met all quality criteria showed a significant effect comparable with the overall effect size. Heterogeneity was low in most analyses, suggesting that most studies pointed in the same direction, with no major outliers. We found some indications for publication bias, but adjusting effect sizes resulted in no major changes.

Effects sizes were slightly lower than those found for cCBT for anxiety and depressive disorders in adults (g = 0.88) [30], were comparable to those found in recent meta-analyses on traditional face-to-face CBT for anxiety disorders in youth (0.66) [15] and somewhat higher as those found for CBT for depression in youth (0.35) [16]. However, we also included studies with mixed samples that also included participants above the age of 18. Removing these studies resulted in an overall ES of 0.61 and NNT of about 2.99, still in range of ESs of face-to-face CBT. The comparable effects to face-to-face psychotherapy are consistent with research on cCBT for psychiatric and somatic disorders in adults, showing no differences between internet-based CBT and face-to-face treatments [59].

We also found significant, moderate but somewhat smaller effects compared to the main-outcome analysis on symptoms of depression when including also outcomes of interventions targeting anxiety. Likewise, we also found significant and moderate effects on symptoms of anxiety when also including outcomes of interventions targeting depression. These findings are consistent with growing evidence indicating that (a) youth depression and anxiety are closely associated [4, 5], (b) psychotherapeutic treatment for youth can have significant effects on comorbid problems [16], and (c) may be treated simultaneously [60]. In fact our results support such an assumption, with significant and large effects (g = 0.94, NNT = 2.02) for those treatments targeting both disorders at the same time [51, 53].

Participant age was the only significant moderator of treatment effects. The finding was consistent also when using different classifications of children (< 13 vs. ≤ 13 years of age). In line with the results of the most recent meta-analyses on psychotherapy for anxiety disorders in youth [15], we found that treatments for children were significant but with smaller effects compared to treatments for adolescents. However, meta-analyses on psychotherapy for depression in youth have not found such an association [16].

We found no association between the inclusion of parents and better treatment outcomes. Although it is often argued that parental involvement is important in the treatment of anxiety and depressive disorders in youth [61], our results are consistent with the meta-analyses of Reynolds and colleagues, showing no additional benefit of treatments with parental involvement [15].

When interpreting results of this study, several limitations should be considered.

First, 11 of 13 studies reviewed reported no follow-up assessment with treatment versus control comparison. Hence, we could not examine long-term effects of treatments. This shortcoming points to the need for future studies with follow-up assessments. Second, the interventions evaluated in the studies were very heterogeneous regarding format of treatment, ranging from therapist focused group-based chat interventions based on face-to-face treatment manuals [57] to unguided more serious gaming-based approaches [45, 53]. Given the large variability and the limited amount of studies available, we could not examine the differential effectiveness of treatment formats. Third, also because of the limited number of studies, we were not able to perform subgroup analyses with adequate power separately for depression and anxiety or other potential subgroup analyses of interest. Fourth, most studies targeting depression excluded participants with severe depression. Consequently, our results should not be generalized to youths with severe depression. Fifths, the present study did not search for unpublished studies. Thus it may be possible that there are unpublished studies that are not included here, which may lead to an over- or under-estimation of the true intervention effect sizes. Sixths, true levels of heterogeneity in meta-analyses cannot be detected with currently available statistical methods [62]. Although we applied a number of state-of-the art methods to estimate heterogeneity (i.e. I² including 95%-CIs, Q statistics, sensitivity analysis excluding studies with the lowest/highest effect size), we cannot rule out that heterogeneity exits and the reported effect sizes are over- or underestimated. Finally, all studies were conducted in high-income countries. The generalizability of the study findings for low- and middle income countries is therefore limited.

Findings from the present study provide evidence for the efficacy of cCBT in the treatment of anxiety and depressive symptoms in youth. Hence, such intervention might be a promising treatment alternative when evidence based face-to-face treatment is not available or simply not wished. Applying cCBT at large scale may thus serve as one (out of several) strategies to bridge the enormous gap between the need and provision of evidence-based treatments for anxiety and depression in children and adolescents. Given that access to evidence-based psychotherapeutic treatment is not only a problem in high, but even more in low- and middle-income countries [54], future studies should evaluate the potential of cCBT effectiveness for youths in non-high income countries. Subsequent studies should also examine the long-term effectiveness of cCBT and evaluate potential negative effects of cCBT [63]. Findings also indicate that more research is necessary to draw clear conclusions with regard to moderators. Finally, this meta-analysis was limited to studies including participants with elevated symptoms, excluding studies directed at the prevention rather than treatment of symptoms (e.g. [64]). Given the potential scalability of Internet-based treatments, there might be a large potential for their use in the prevention of common mental health disorders [65, 66], which should also be examined in future studies.

## Supporting Information

S1 PRISMA Checklist PRISMA Checklist. (DOC)

S1 Text Review Protocol. (DOC)
