# Supplementary material for: Internet and Computer-Based Cognitive Behavioral Therapy for Anxiety and Depression in Youth: A Meta-Analysis of Randomized Controlled Outcome Trials
Source: PLoS One. 2015 Mar 18;10(3):e0119895. doi: 10.1371/journal.pone.0119895 (PMC4364968; doi:10.1371/journal.pone.0119895)
Supplement: S1 Text — (DOC) [file pone.0119895.s002.doc]

**Text S1 Study protocol for a meta-analyses to evaluate the effects of cCBT in the treatment of anxiety and depression in youth.**

**Objective**

To examine the effects of cCBT for anxiety and depression in youth (up to the age of 25) in comparison to a non-active control condition within randomized controlled trials.

**Reporting**

We will adhere to the PRISMA (Preferred Reporting Items for Systematic Reviews and Meta-Analyses) statement for reporting results.

**Search Methods**

We will systematically search Pubmed, Cochrane library for randomized trials and PsychInfo for relevant articles. We will also check references of relevant articles. We will not apply any language restrictions. Our search will be performed until December, 4th, 2013.

Our database search will include the terms (Internet OR web OR Computer OR CD-ROM OR virtual reality OR online) AND (adolescent OR Youth OR children OR young adults) AND (Intervention OR Psychotherapy OR prevention OR treatment) AND (depression OR Anxiety OR transdiagnostic OR Phobia).

We will use full-text search as well as meSH terms.

Depending on the database the search will be restricted to: Human & Methodology: Treatment Outcome / Clinical Trial & Age Groups: <18 – 25 & Treatment & Prevention:

**Eligibility**

*Inclusion criteria*

The following inclusion criteria will be applied:

Studies will be only included if

1. They applied a randomized controlled design
2. If they evaluated a computer/internet/mobile-based treatment directed at the treatment of anxiety, depression or both
3. If the treatment was based on CBT
4. If the treatment was compared to a non-active control condition (waitlist, no-treatment, attention control)
5. If it was evaluated in a sample up to the age of 25 years (studies in which all participants were above 18 years of age will be excluded)
6. If the primary outcome was either depression, anxiety or both.
7. If participants were selected based on elevated symptoms of depression, anxiety or both (studies that did not apply either a cut-off score based on an standardized instrument or diagnostic interview will be excluded)
8. If effect sizes can be calculated from the data extracted from the paper or if authors provide necessary data for the effect size calculation.
9. The Internet/computer was the primarily treatment modality (studies which evaluated interventions primarily based on face-to-face psychotherapy and including some online-elements will be excluded)

**Data Collection**

Two reviewers (DDE, AZ) will independently examine studies identified through searches. Both reviewers will base final selection on the full text of potentially relevant articles. A third reviewer (HR) will be consulted to determine eligibility of articles in cases of disagreement. Study, participant and intervention characteristics from all eligible studies will be also extracted by two independent reviewers (DDE and AZ). Inter-rater reliability will be calculated for agreements between raters.

Data for calculating effect size will be entered in comprehensive meta-analyses software package by a single reviewer (DDE).

**Assessing risk of Bias**

Risk of bias assessment will be again conducted independently by two assessors (DDE & AZ) Assessement of the validity of included studies will be based on four criteria of the *Risk of Bias* assessment tool, developed by the Cochrane Collaboration (adequate generation of allocation sequence, the concealment of allocation to conditions, masking of assessors, and the missing data handling. Missing data handling will be rated as positive when intention-to-treat analyses were conducted. To determine whether the risk of bias score is associated with the effect size, we will apply meta-regression with the risk of bias score as predictor and the effect size as dependent variable. We well also compare in subgroup analyses the effect size of studies fulfilling all assessed criteria with studies fulfilling less than four criteria.

**Study coding**: Characteristics of all included studies will be extracted and classified to the following criteria:

Study Design: Target condition (depression, anxiety, transdiagnostic); comparison condition (no-treatment, wait-list; attention/placebo control); Outcome Informant (Youth, parent, teacher); Outcome Assessment (Self-report, observer-based); Follow-Up Assessment (Yes/No, Duration of follow-up).

Participant characteristics: mean age and age group (child < 1, years of age; adolescents > 13 years of age; mixed: both children and adolescents); percentage of boys included in the study; inclusion of participants based on diagnostic interview (yes/no).

**Intervention characteristics:** Parental involvement (yes/no) guidance (therapeutic, administrative, no guidance); delivery mode (computer; Internet; mobile), treatment setting (home /clinic/school, number of treatment sessions.

**Meta-analysis**

To calculate effect sizes, we will use Comprehensive Meta-Analysis (CMA). For the main outcome analyses we will pool effect sizes of all individual studies based on outcome instruments related to the principal measure of the disorder. For transdiagnostic interventions we will pool effect size of depression and anxiety outcomes. In secondary analyses we will also examine the overall effects on depression and anxiety respectively, including also secondary outcomes. If depression and anxiety was assessed with more than one measure (e.g. self-report and obserserverbased rating) we will pool the effect sized across measures, so that each study provide only one effect size. To estimate the size of the effect we will calculate hedges g (using CMA) and will also transform these value to Numbers-needed-to-be-treated. We expect substantial heterogeneity, hence we will employed a random-effects model. Subgroup analyses will we conducted using a mixed-effect model. I² and its 95% confidence interval will be calculated to assess heterogeneity and we also will calculate Q statistics.

Publication bias: To assess a potential of bias we will a) inspect the funnel plot b) conduct Egger’s test and c) use the The Duval and Tweedie trim-and-fill analysis to estimate the size of the intervention effects, when taking publication bias into account.
